# LncRNA PFAR facilitates the proliferation and migration of papillary thyroid carcinoma by competitively binding to miR-15a

**DOI:** 10.1007/s00210-023-02779-w

**Published:** 2023-10-24

**Authors:** Tie Fang, Kejie Yu

**Affiliations:** Department of Thyroid Surgery, Ningbo No. 2 Hospital, No.41, Northwest Street, Haishu District, Ningbo City, 315000 Zhejiang Province China

**Keywords:** Papillary thyroid carcinoma, LncRNA PFAR, miR-15a, RET, AKT/mTOR signaling

## Abstract

Papillary thyroid carcinoma (PTC) is type of aggressive tumor, with a markedly declined survival rate when distant metastasis occurs. It is of great significance to develop potential biomarkers to evaluate the progression of PTC. LncRNAs are recently widely claimed with biomarker value in malignant tumors. Herein, the role of LncRNA PFAR in PTC was investigated to explore potential prognostic marker for PTC. Compared to NTHY-ORI 3-1 cells, LncRNA PFAR was found markedly upregulated in PTC cell lines. In LncRNA PFAR knockdown TPC-1 cells, markedly declined cell viability, increased apoptotic rate, enhancive number of migrated cells, and elevated migration distance were observed, accompanied by a suppressed activity of the RET/AKT/mTOR signaling. In LncRNA PFAR overexpressed BCPAP cells, signally increased cell viability, declined apoptotic rate, reduced number of migrated cells, decreased migration distance, and increased tumor volume and tumor weight in nude mice xenograft model were observed, accompanied by an activation of the RET/AKT/mTOR signaling. The binding site between LncRNA PFAR and miR-15a, as well as miR-15a and RET, was confirmed by the dual luciferase reporter assay. The FISH study revealed that LncRNA PFAR was mainly located in the cytoplasm. Furthermore, the impact of the siRNA targeting LncRNA PFAR against the growth and migration of PTC cells was abolished by the inhibitor of miR-15a or SC79, an activator of AKT/mTOR signaling. Collectively, LncRNA PFAR facilitated the proliferation and migration of PTC cells by mediating the miR-15a/RET axis.

**Supplementary Information:**

The online version contains supplementary material available at 10.1007/s00210-023-02779-w.

## Introduction

Thyroid carcinoma (TC) is one of the most common endocrine diseases in women, which is influenced by multiple factors, such as genetics, environment, immunosuppression, and unscientific diet. TC is a malignant tumor with the fastest increasing incidence in the past two decades, and also one of the main causes of death (Hu et al. 2021; Haddad et al. 2022). TC is divided into four main histological types, which were PTC, follicular thyroid carcinoma (FTC), medullary thyroid carcinoma (MTC), and anaplastic thyroid carcinoma (ATC) (Asa 2019). Among them, PTC accounts for about 80.85% of all TC cases, occupying an absolute dominant position (Zhang et al. 2020; Xu et al. 2020). Although PTC is a low-grade tumor, and the overall 5-year survival rate of patients with PTC after effective treatment is generally higher than 95% (Tong et al. 2020), PTC will be developed into aggressive cancer in the presence of distant metastasis, with a reduced survival to about 50% (Pinto et al. 2011). Therefore, it is important to investigate the migration process and molecular mechanism of PTC cells to further improve the survival rate of PTC patients.

With the rapid development of high-throughput sequencing technology, researchers have discovered a class of long non-coding RNAs (LncRNAs) with a length of more than 200 nucleotides, which are localized in the nucleus or cytoplasm. By mediating the gene expression at the level of chromatin transcription, modification, and post-transcriptional expression (Park et al. 2022; Lin et al. 2020), LncRNAs are involved in the regulation of cell development, migration, pluripotency, cycle, apoptosis, and invasion (Lin et al. 2020). It is confirmed that LncRNAs play a critical role in the development of human diseases, especially malignant tumors (Yang et al. 2022; Bridges et al. 2021). LncRNAs are abnormally expressed in a variety of tumors and closely related to the changes of tumor biological behavior, which are involved in tumor invasion, metastasis, autophagy, differentiation and other biological processes (Entezari et al. 2022). In PTC, LncRNAs are also reported to act as "oncogenes" or "tumor suppressor genes" (Mahmoudian-Sani et al. 2019; Sedaghati and Kebebew 2019; Zheng et al. 2016). MicroRNAs (miRNAs) were first discovered in Caenorhabditis elegans, and later similar non-coding endogenous small RNAs were found in different organisms (Gierlikowski and Gierlikowska 2022; Pozniak et al. 2022). MiRNAs are highly conserved, with 18–22 nucleotides length (Komatsu et al. 2023), and play a role in RNA silencing and post-transcriptional regulation of gene expression by binding to the 3 'Untranslated regions (UTR) of target genes, leading to degradation or inhibition of transcription of target genes (Jonas and Izaurralde 2015). MiRNA regulates a variety of cellular processes, including proliferation, apoptosis, differentiation, and metastasis (Zhao et al. 2022). Studies have found that the dysregulation of miRNA is related to a variety of human malignant tumors. Similar to LncRNAs, miRNAs also act as "oncogenes" or "cancer suppressor genes" (Zhang et al. 2007). By affecting tumor invasion and migration of PTC, miRNAs may be used as prognostic biomarkers for PTC (Wei et al. 2015; Zhang et al. 2014; Wang et al. 2013). Recently, it is claimed that miR-15a is downregulated in PTC tissues compared to normal tissues and inhibits the PTC cell proliferation and invasion (Jin et al. 2019). LncRNA PFAR is reported to target miR-15a and regulates its expression (Sun et al. 2019). However, the function of LncRNA PFAR in PTC remains uncertain. Herein, the role of LncRNA PFAR-miR-15a axis in PTC was investigated to explore potential prognostic markers for PTC.

## Materials and methods

### Cells and treatments

Human thyroid normal cells (NTHY-ORI 3-1 cells) and PTC cell lines (BHP5-16, BCPAP, TPC-1, and K1 cells) were obtained from iCell (China) and cultured in DMEM medium supplemented with 10% FBS, which were cultured at 37℃ and 5% CO2.

### Transfection

To knockdown LncRNA PFAR in TPC-1 cells, cells were transfected with siRNAs (siR-PFAR-1, siR-PFAR-2, and siR-PFAR-3) targeting PFAR under the assistance of lipofectamine 3000 (Invitrogen, USA) for 48 h, followed by evaluating the transfection efficacy using RT-PCR to screen the optimized siRNA. SiR-NC was taken as the negative control. To obtain the LncRNA PFAR-overexpressed BCPAP cells, the overexpression-vector of LncRNA PFAR (pcDNA3.1-PFAR) was imported into lentiviral particles, which were transfected into BCPAP cells for 48 h, followed by evaluating the transfection efficacy using RT-PCR assay. pcDNA3.1-NC was taken as the negative control.

### RT-PCR assay

Total RNAs were obtained from cells using the TRIzol reagent and were quantified with the Nanodrop one (Thermo Scientific, USA). A cDNA synthesis kit (CW2569, CWBIO, China) was utilized for conducting the transcription from RNAs to cDNAs, followed by performing the PCR reaction utilizing a SYBR Green kit (11201ES08, YEASEN, China). The 2^−ΔΔCt^ method was used for the calculation of gene levels. Sequences of primers were shown in Table 1. For the detection of LncRNA PFAR level, GAPDH or RNU6 snRNA was taken as the negative control. For the detection of miR-15a level, U6 was taken as the negative control.

**Table 1:** Sequences of primers in the RT-PCR assay

Gene	Forward Primer	Reverse Primer
LncRNA PFAR	CCTTGGAGTAAAGTAGCAGCAC	GAGGCAGCACAATATGGCCT
miR-15a	CTTGGAGTAAAGTAGCAGCACAT	AGGCAGCACAATATGGCCT
U6	AAAGCAAATCATCGGACGACC	GTACAACACATTGTTTCCTCGGA
RNU6 snRNA	GGAACGATACAGAGAAGATTAGC	TGGAACGCTTCACGAATTTGCG
GAPDH	GGAGCGAGATCCCTCCAAAAT	GGCTGTTGTCATACTTCTCATGG

### CCK-8 assay

Transfected cells were digested, resuspended, counted, and plated at a cell density of 6 × 10^3^ cells/well. After adhesion, 10 μL of CCK8 reagent was added to each well and incubated in the incubator for 2 h. The absorbance value of each well was measured at a wavelength of 450 nm by a microplate reader (CMaxPlus, MD, USA).

### Apoptosis detection using the flow cytometry

Cells of each group were placed in a constant temperature incubator for 48 h, followed by resuspended and adding 10 μL Annexin V reagent and 5 μL PI reagent. Cells were incubated at room temperature for 10 min, and the appropriate amount of cell suspension was sucked and mixed with PBS in flow assay tubes, followed by loading onto the flow cytometry (NovoCyte, Agilent, USA) for apoptosis detection.

### Transwell study for migration detection

500 μL of complete medium was added to the lower chamber. The cell suspension was added to the upper chamber and incubated for 12 h at 37℃ and 5% CO2 in the medium without serum. The upper chamber medium was discarded, and the Transwell chambers were washed twice with PBS at room temperature, followed by transferred to the well of a 24-well plate pre-added with 700 μl 4% formaldehyde. After fixing for 20 min, 700 μl of 0.1% crystal violet was added and washed. The images were taken under an inverted microscope (AE2000, Motic, China), with five fields counted.

### Wound healing assay

Cells (2 × 10^5^ cells /mL) were seeded in 6-well plates. After reaching 80%-90% density, cells were scraped with a yellow pipet, and the scratch area was washed twice with PBS to remove floating cells. The scratch width was recorded at 0 h. After continuous incubation for 48 h in medium containing 5% serum, the wound was photographed again and the scratch width and wound healing rate were calculated.

### Western blotting assay

Total proteins were extracted from cells and quantified with the BCA method, which were separated using the 12% SDS-PAGE, followed by transferred to the PVDF membrane. Following blocking, primary antibodies against RET (1:1000, AF6120, Affinity, USA), p-AKT (1:1000, AF0016, Affinity, USA), AKT (1:1000, AF6259, Affinity, USA), mTOR (1:1000, AF6308, Affinity, USA), p-mTOR (1:1000, AF3308, Affinity, USA), and GAPDH (1:10000, 1094-1-AP, Proteintech, USA) were added to be cultured for 12 h at 4 ℃, followed by incubation with the secondary antibody (1:6000, 7074, CST, USA) and incubated for 60 min. Lastly, the ECL solution was added for exposure and the expression level was quantified with the Image J software.

### Xenograft model

18 female nude mice were divided into 3 groups: Control, pcDNA3.1-NC, and pcDNA3.1-PFAR. Nude mice were subcutaneous implanted with BCPAP cells (1 × 10 ^7^/mice) transfected with pcDNA3.1-NC and pcDNA3.1-PFAR, respectively, with nude mice implanted with normal BCPAP cells as the control group. The tumor volume was measured every 3 days (7 times in total), and the tumor tissue was weighed on the 21^st^ day post implantation.

### Dual luciferase reporter assay

Cells were seeded in 24-well plates at a density of 5 × 10^4^ cells/well. The next day, 200 ng of pMir-reporter fluorescent vector containing the 3' UTR region of LncRNA PFAR or RET and wild-type miR-15a, 200 ng of pMir-reporter fluorescent vector containing the 3' UTR of LncRNA PFAR or RET and mutant miR-15a, 200 ng miR-15a, and 200 ng negative control oligonucleotide (miR-Ctrl) were transfected into 293 T cells (ATCC, USA) using lipofectamine 3000 (Invitrogen, USA). After 48 h, the luciferase activity of each group was detected by dual luciferase system (RG027, Beyotime, China).

### Fluorescence in situ hybridization (FISH)

Cells were fixed in 4% paraformaldehyde for 15 min at room temperature and then permeabilized in precooled PBS containing 0.5% Triton X-100 for 5 min. After washed three times for 10 min each in PBS and rinsed once in 2 × SSC buffer, hybridization was performed with probes for LncRNA PFAR, U6, and 18S for 12 to 16 h at 37 ℃. Cells were then washed with 4 × SSC buffer, 2 × SSC buffer, and 1 × SSC buffer, respectively. Finally, cells were counterstained with DAPI for 10 min and visualized by laser confocal microscopy (LSM880, ZEISS, Germany).

### Statistical analysis

Data were expressed using mean ± SD and analyzed with the one-way ANOVA method followed by Tukey’s test using the GraphPad prism software 6.0. *P* < 0.05 was considered a significant difference.

## Results

### LncRNA PFAR was upregulated in PTC cells and the transfection in TPC-1 cells and BCPAP cells was identified

**Fig. 1:**
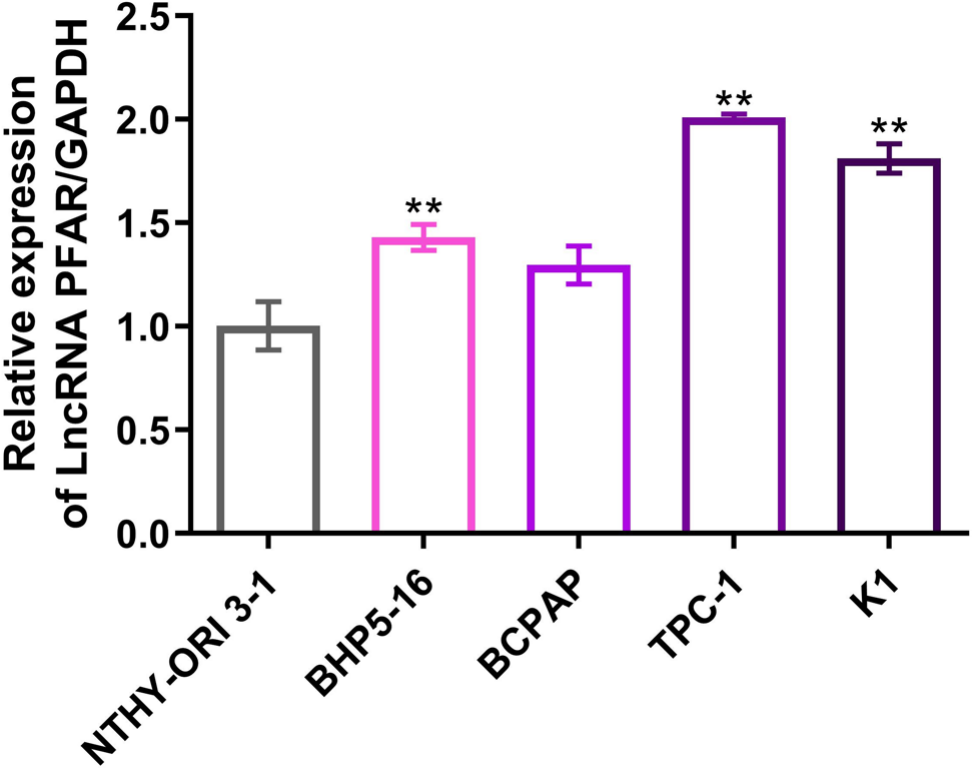
LncRNA PFAR was upregulated in PTC cell lines. RT-PCR assay was utilized to determine the LncRNA PFAR level in NTHY-ORI 3-1 cells, BHP5-16, BCPAP, TPC-1, and K1 cells (**p < 0.01 vs. NTHY-ORI 3-1 cells, *n* = 3)

**Fig. 2:**
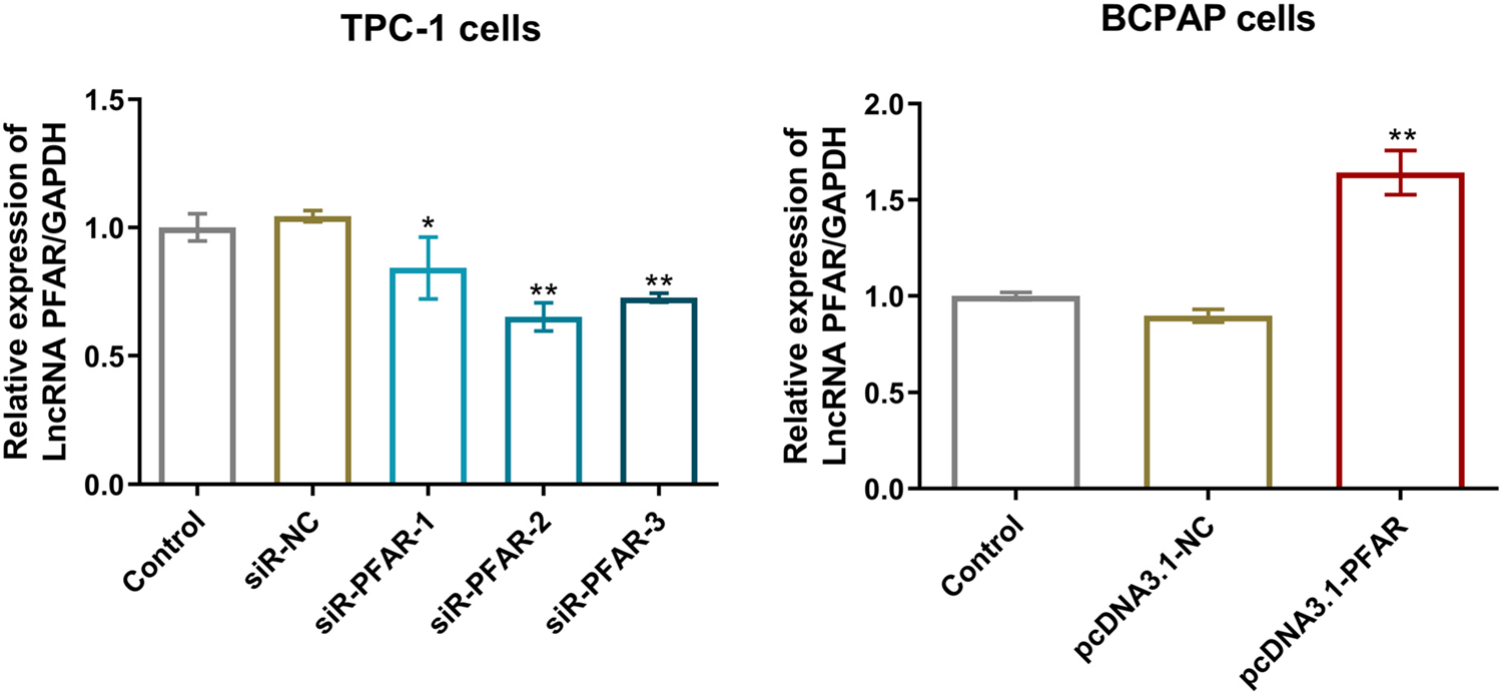
LncRNA PFAR was successfully knocked down in TPC-1 cells and overexpressed in BCPAP cells. The level of LncRNA PFAR level in TPC-1 cells and BCPAP cells was detected by the RT-PCR assay after transfection (**p < 0.01 vs. siR-NC or pcDNA3.1-NC, *n* = 3)

Firstly, the level of LncRNA PFAR in human thyroid normal cells and human PTC cells were compared. Compared to NTHY-ORI 3-1 cells, the LncRNA PFAR was found markedly increased in BHP5-16, TPC-1, and K1 cells, and slightly elevated in BCPAP cells with no significant difference (Fig. 1, Fig. S1A). To explore the potential function of LncRNA PFAR in PTC, LncRNA PFAR was knocked down in TPC-1 cells, in which highest level of LncRNA PFAR was observed. Furthermore, LncRNA PFAR was overexpressed in BCPAP cells. The knockdown efficacy was evaluated by RT-PCR and siR-PFAR-2 with the highest transfection efficacy was chosen for subsequent assays. Furthermore, LncRNA PFAR was found markedly upregulated in pcDNA3.1-PFAR transfected BCPAP cells (Fig. 2, Fig. S1B-C).

### LncRNA PFAR facilitated the proliferation and suppressed the apoptosis of PTC cells

**Fig. 3:**
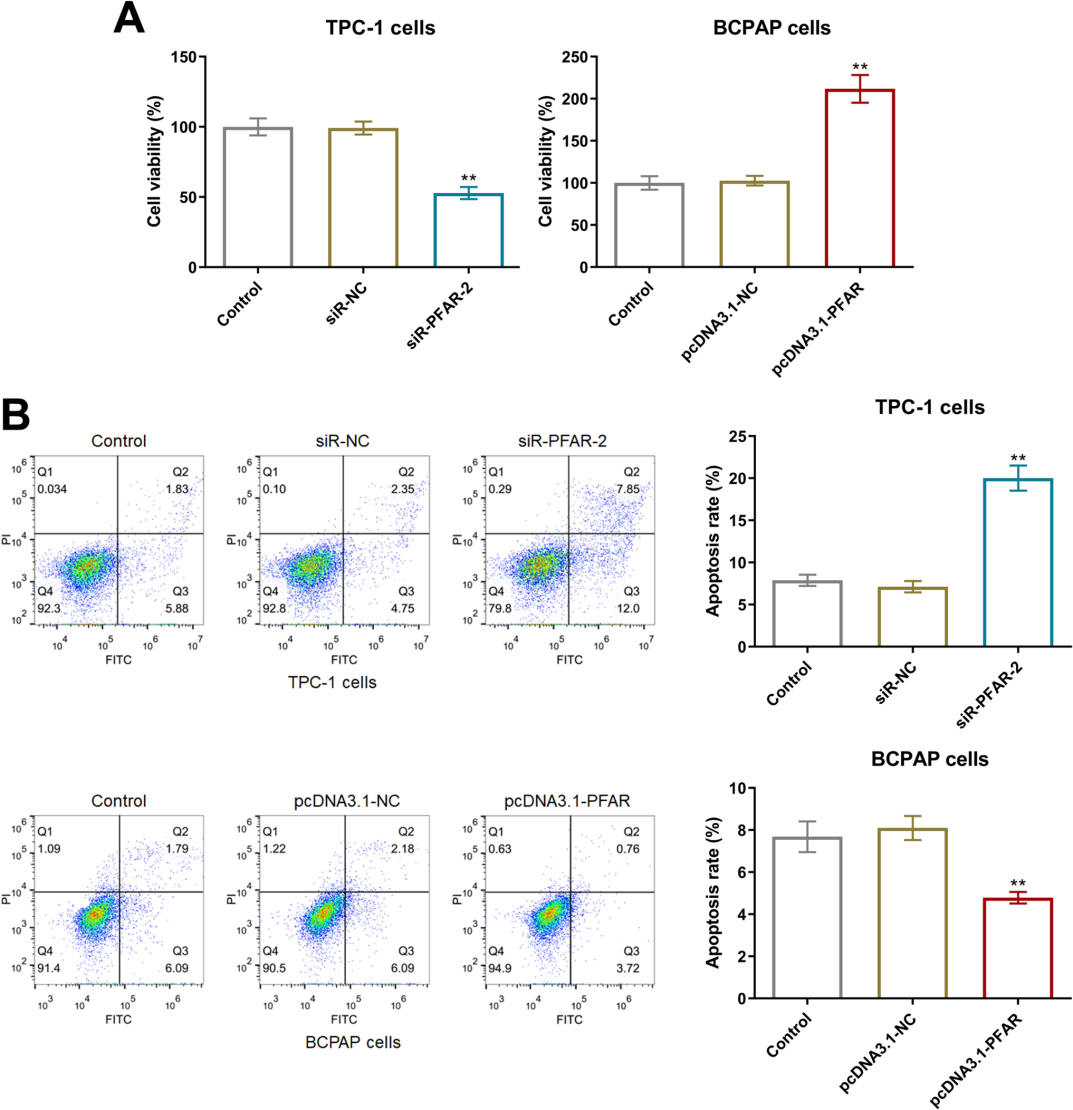
The proliferation was facilitated and the apoptosis was inhibited by LncRNA PFAR in PTC cells. **A** The cell viability of TPC-1 cells and BCPAP cells was measured by CCK-8 assay (*n* = 6). **B** The apoptotic rate of TPC-1 cells and BCPAP cells was detected by the flow cytometry (**p < 0.01 vs. siR-NC or pcDNA3.1-NC, *n* = 3)

The cell viability of TPC-1 cells was slightly changed from 100% to 99.2% by siR-NC and was sharply reduced to52.9% by siR-PFAR-2. Furthermore, in BCPAP cells, the cell viability in the control, pcDNA3.1-NC, and pcDNA3.1-PFAR groups was 100%, 102.7%, and 211.8%, respectively (Fig. 3A). In TPC-1 cells, the apoptotic rate in the control, siR-NC, and siR-PFAR groups was 7.61%, 7.10%, and 19.85%, respectively. Moreover, the apoptotic rate in BCPAP cells was minorly changed from 7.88% to 8.27% by pcDNA3.1-NC and markedly reduced to 4.48% by pcDNA3.1-PFAR (Fig. 3B).

### LncRNA PFAR enhanced the migration ability of PTC cells

**Fig. 4:**
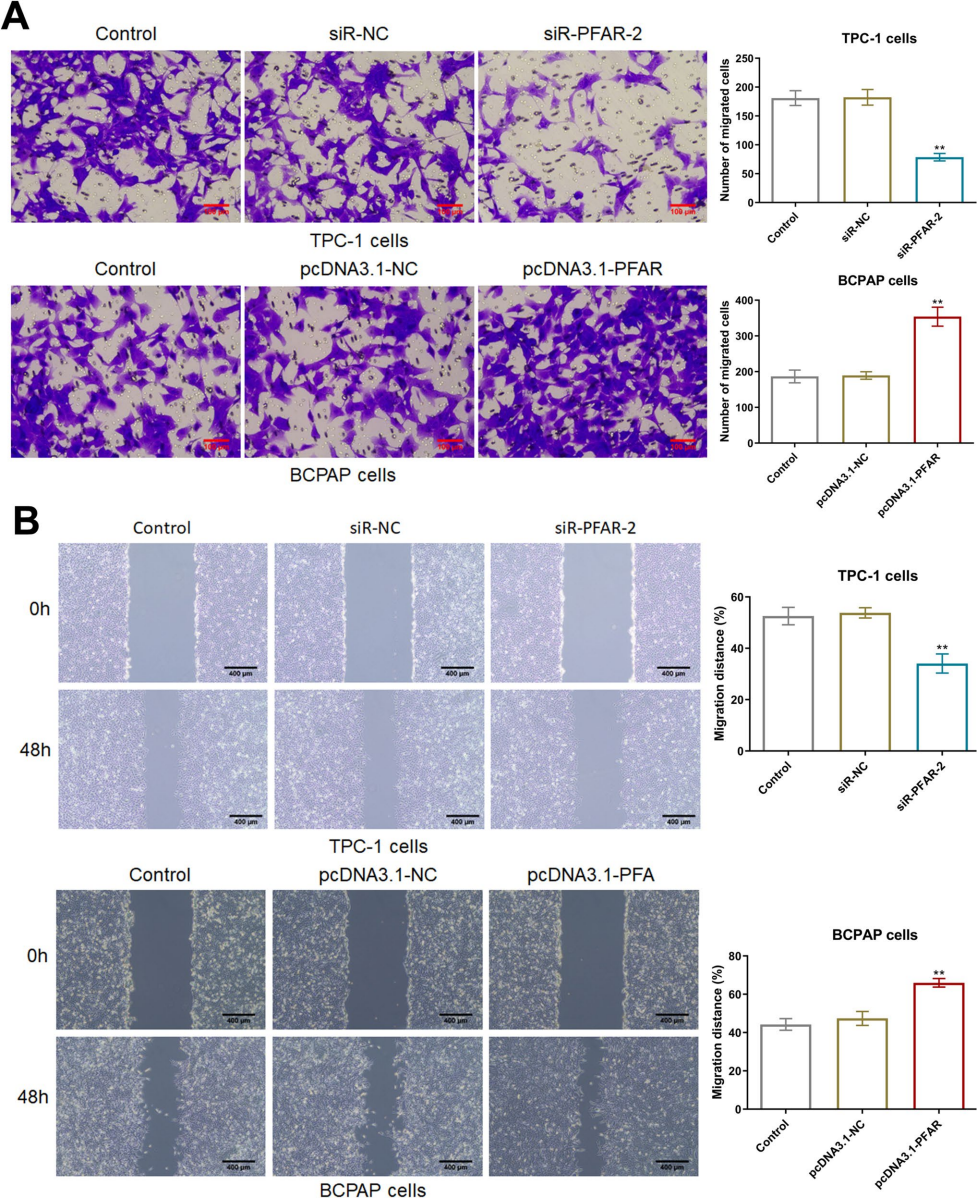
The migration of PTC cells was enhanced by LncRNA PFAR. **A** The migration ability of TPC-1 cells and BCPAP cells was determined by Transwell assay (*n* = 3). **B** The wound healing assay was utilized to detect the metastasis of TPC-1 cells and BCPAP cells (**p < 0.01 vs. siR-NC or pcDNA3.1-NC, *n* = 3)

Subsequently, the migration ability of TPC-1 cells and BCPAP cells were determined. The number of migrated TPC-1 cells was slightly changed from 181.0 to 182.3 by siR-NC and signally reduced to 78.7 by siR-PFAR-2. However, in BCPAP cells, the number of migrated cells in the control, pcDNA3.1-NC, and pcDNA3.1-PFAR groups was 186.7, 189.3, and 354.0, respectively (Fig. 4A). Furthermore, in the wound healing study, the migration distance of TPC-1 cells in the control, siR-NC, and siR-PFAR groups was 52.6%, 53.8%, and 34.1%, respectively. The migration distance of BCPAP cells was minorly altered from 44.3% to 47.4% by pcDNA3.1-NC and largely increased to 66.0% by pcDNA3.1-PFAR (Fig. 4B).

### LncRNA PFAR repressed the miR-15a level and activated the RET/AKT/mTOR signaling in PTC cells

**Fig. 5:**
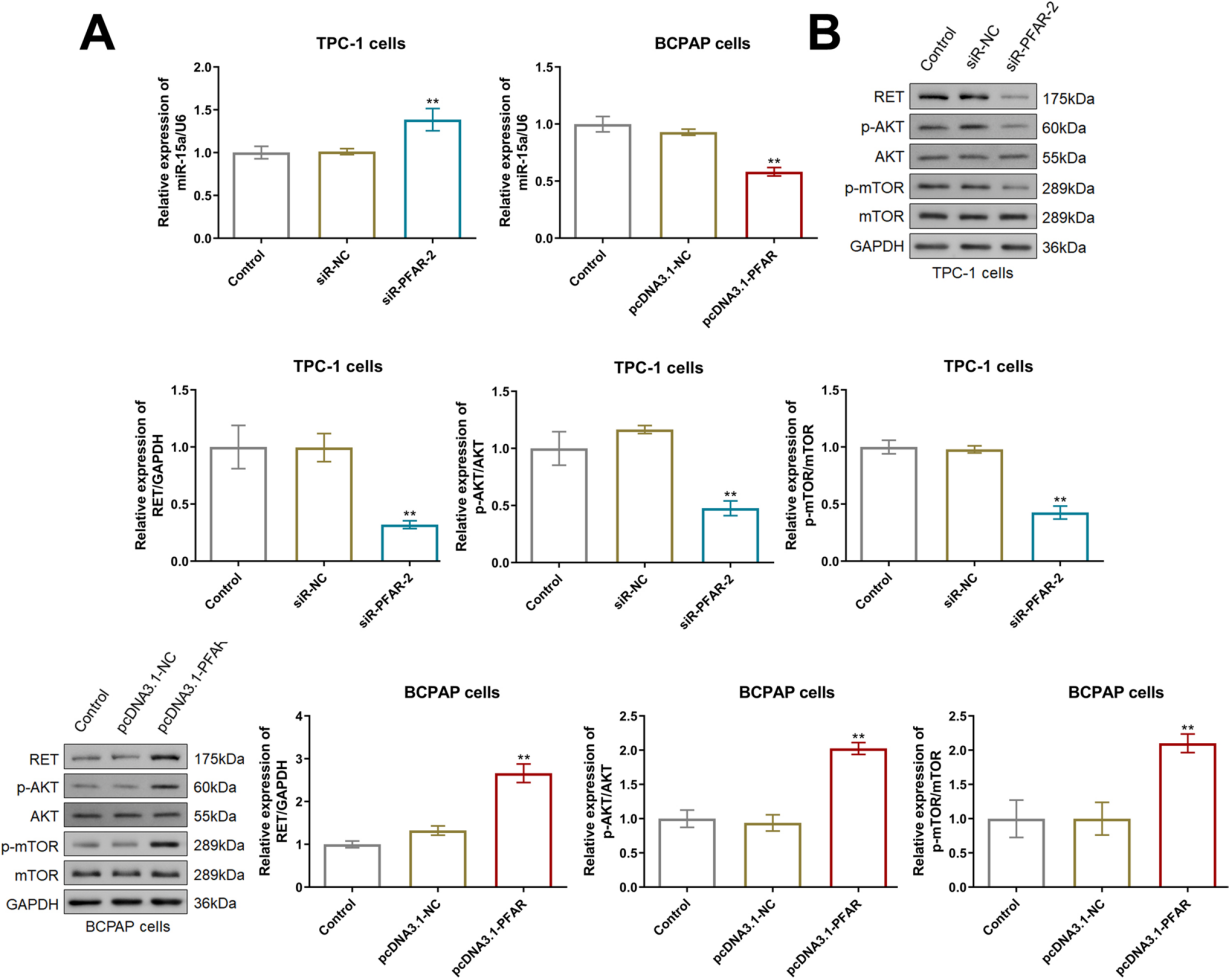
LncRNA PFAR repressed the miR-15a level and activated the RET/AKT/mTOR signaling in PTC cells. The expression level of RET, p-AKT, AKT, p-mTOR, and mTOR was detected by Western blotting assay (**p < 0.01 vs. siR-NC or pcDNA3.1-NC, *n* = 3)

As shown in Fig. 5A, in TPC-1 cells, the miR-15a level was found markedly increased by siR-PFAR, while in BCPAP cells, the miR-15a level was signally reduced by pcDNA3.1-PFAR. Furthermore, in TPC-1 cells, compared to siR-NC, levels of RET, p-AKT/AKT, and p-mTOR/mTOR were sharply repressed by siR-PFAR-2. In BCPAP cells, compared to pcDNA3.1-NC, levels of RET, p-AKT/AKT, and p-mTOR/mTOR were notably elevated by pcDNA3.1-PFAR (Fig. 5B).

### LncRNA PFAR facilitated the in vivo growth of PTC cells

**Fig. 6:**
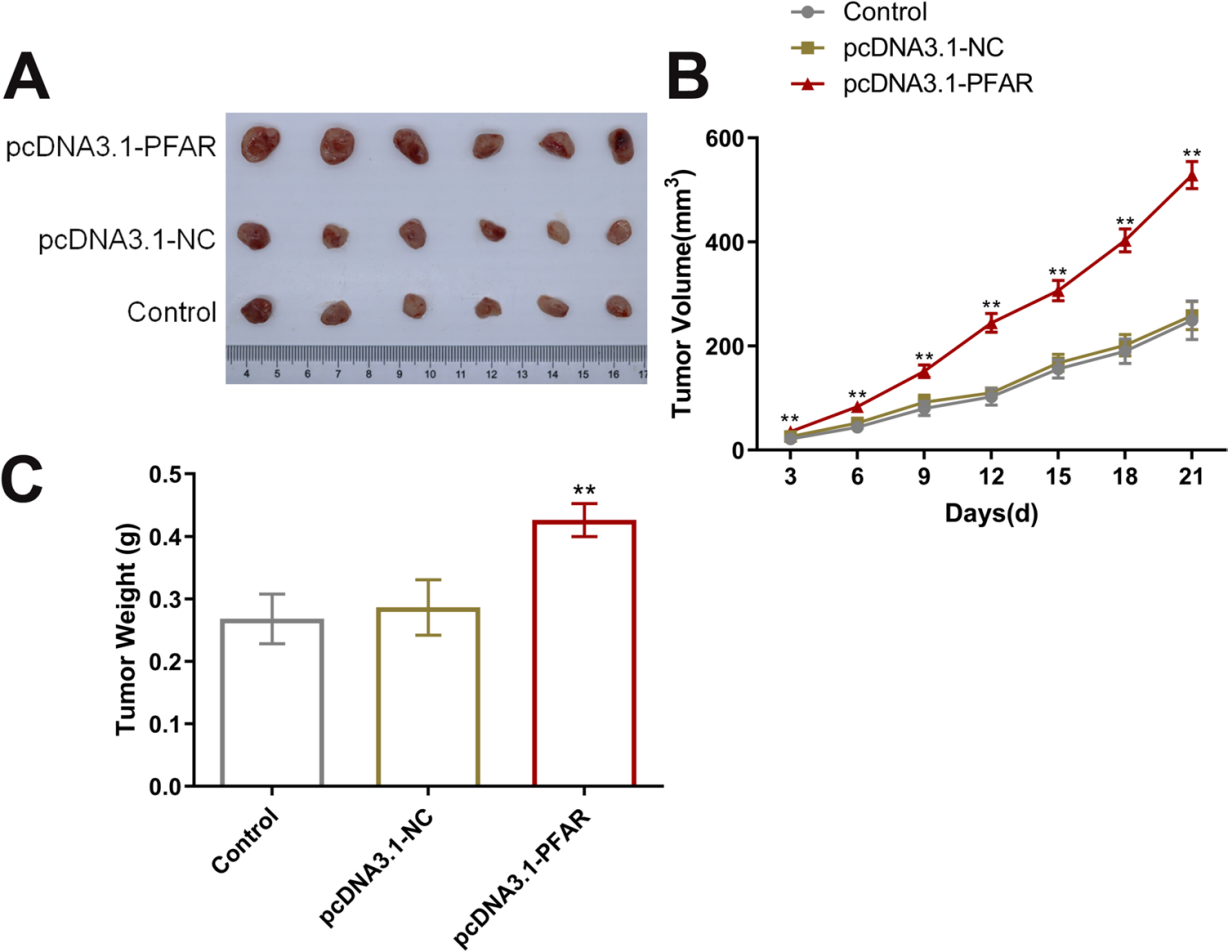
LncRNA PFAR facilitated the in vivo growth of BCPAP cells in the xenograft model of nude mice. **A** Images of tumors isolated from nude mice (*n* = 6). **B** The volume of tumor tissues was recorded (*n* = 6). **C** The tumor weight was recorded (**p < 0.01 vs. pcDNA3.1-NC, *n* = 6)

To confirm the function of LncRNA PFAR, Nude mice were subcutaneous implanted with BCPAP cells transfected with pcDNA3.1-NC and pcDNA3.1-PFAR, respectively, with nude mice implanted with normal BCPAP cells as the control group. Images of tumors were shown in Fig. 6A. Compared to the pcDNA3.1-NC group, the tumor volume (Fig. 6B) and tumor weight (Fig. 6C) were markedly increased in the pcDNA3.1-PFAR group.

### LncRNA PFAR upregulated the RET level by sponging miR-15a

**Fig. 7:**
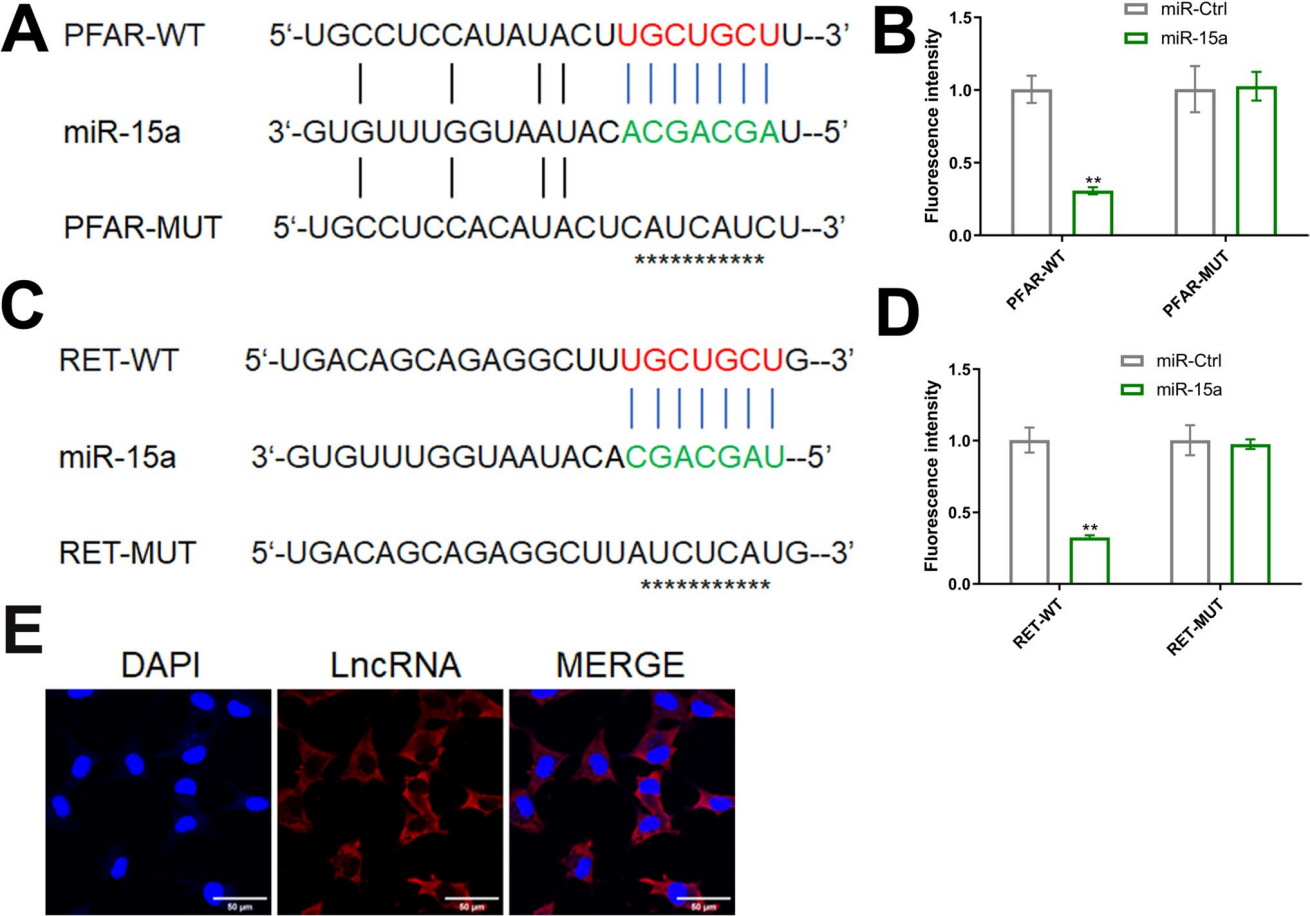
LncRNA PFAR upregulated the RET level by sponging miR-15a. **A** The binding site between LncRNA PFAR and miR-15a was predicted. **B** The binding between LncRNA PFAR and miR-15a was confirmed by the dual luciferase reporter assay (*n* = 3). **C** The binding site between the 3’UTR region of RET and miR-15a was predicted. **D** The binding between the 3’UTR region of RET and miR-15a was confirmed by the dual luciferase reporter assay (**p < 0.01 vs. miR-Ctrl, *n* = 3). **E** The location of LncRNA PFAR was visualized using the FISH study (*n* = 3)

The binding site between LncRNA PFAR (red) and miR-15a (green) was shown in Fig. 7A. In the PFAR-WT group, compared to miR-Ctrl, the fluorescence intensity was markedly declined by miR-15a, while in the PFAR-MUT group, no significant difference on the fluorescence intensity was observed between the miR-Ctrl and miR-15a group (Fig. 7B). The binding site between the 3’UTR region of RET (red) and miR-15a (green) was shown in Fig. 7C. In the RET-WT group, compared to miR-Ctrl, the fluorescence intensity was signally repressed by miR-15a, while in the RET-MUT group, no significant difference on the fluorescence intensity was observed between the miR-Ctrl and miR-15a group (Fig. 7D). Furthermore, the FISH study revealed that LncRNA PFAR was mainly located in the cytoplasm (Fig. 7E).

### LncRNA PFAR facilitated the growth and migration of PTC cells by activating RET/AKT/mTOR signaling via sponging miR-15a

**Fig. 8:**
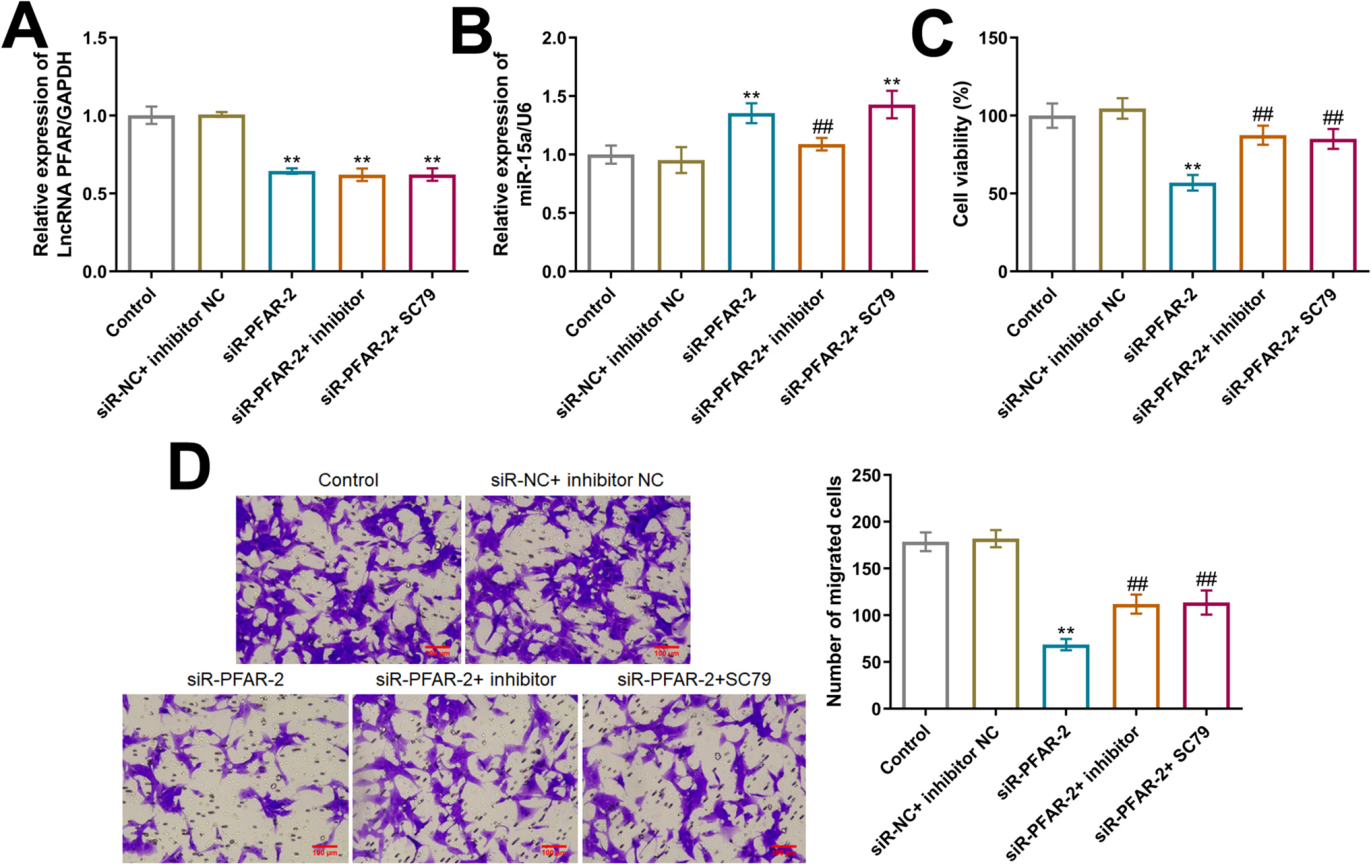
The influence of LncRNA PFAR on the proliferation and migration of PTC cells was abolished by miR-15a inhibitor or SC79. **A** The level of LncRNA PFAR was determined by the RT-PCR assay (*n* = 3). **B** The level of miR-15a was determined by the RT-PCR assay (*n* = 3). **C** The cell viability of TPC-1 cells was measured by CCK-8 assay (*n* = 6). **D** The migration ability of TPC-1 cells was determined by Transwell assay (**p < 0.01 vs. siR-NC+ inhibitor NC, ## *p* < 0.01 vs. siR-PFAR-2, *n* = 3)

**Fig. 9:**
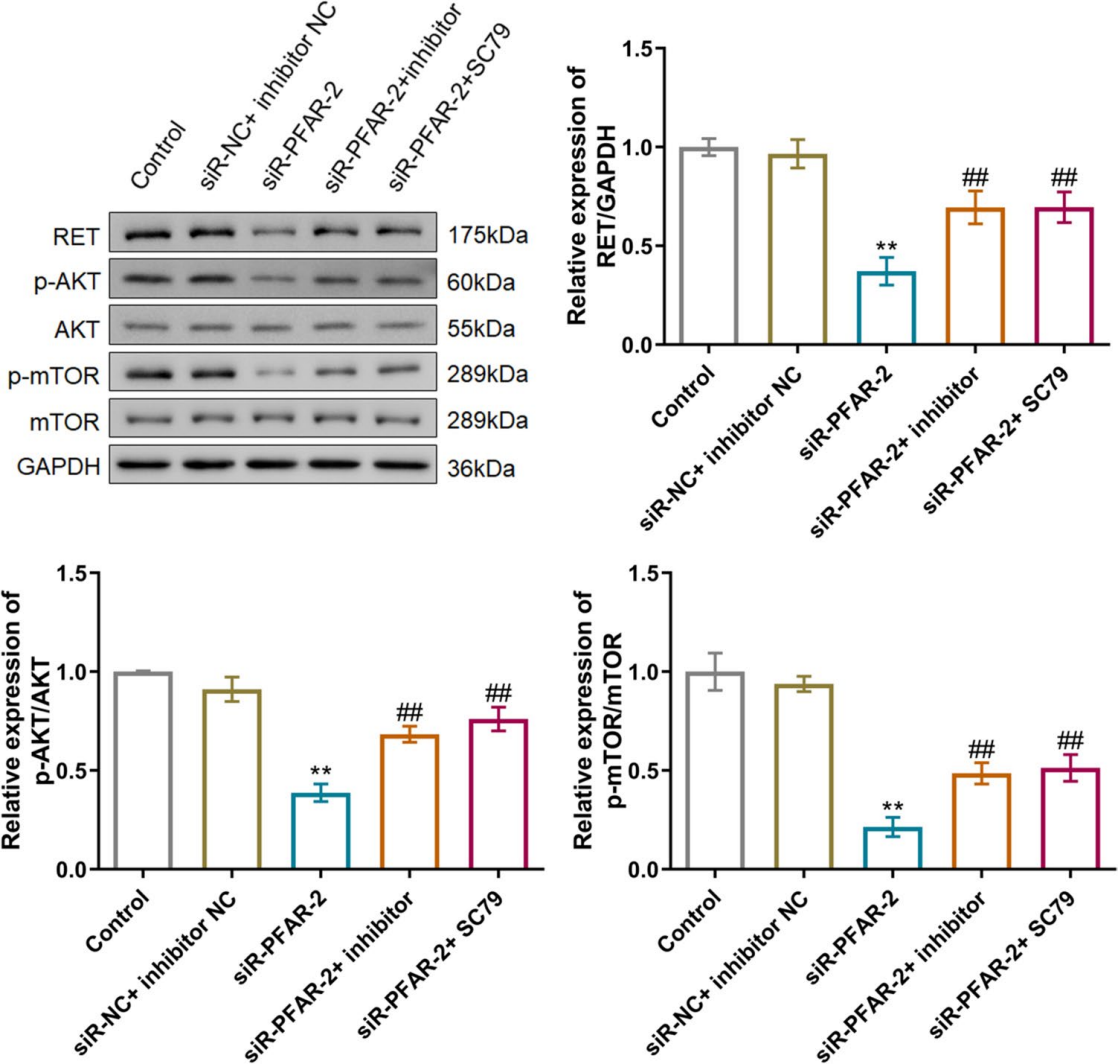
The impact of LncRNA PFAR on the RET/AKT/mTOR signaling was abolished by miR-15a inhibitor or SC79. The expression level of RET, p-AKT, AKT, p-mTOR, and mTOR was detected by Western blotting assay (**p < 0.01 vs. siR-NC+ inhibitor NC, ## *p* < 0.01 vs. siR-PFAR-2, *n* = 3)

To verify the functional mechanism of LncRNA PFAR in PTC, TPC-1 cells were transfected with siR-PFAR-2 in the presence or absence of miR-15a inhibitor or 10 µM SC79 (an activator of AKT signaling). Compared to the siR-NC+ inhibitor NC group, the level of LncRNA PFAR was markedly declined in the siR-PFAR-2, siR-PFAR-2+ inhibitor, and siR-PFAR-2+ SC79 groups (Fig. 8A, Fig. S1D). Furthermore, compared to the siR-NC+ inhibitor NC group, the level of miR-15a was sharply increased by siR-PFAR-2, which was signally reduced in the siR-PFAR-2+ inhibitor group (Fig. 8B). The cell viability was largely reduced from 104.6% to 56.9% by siR-PFAR-2, which was signally increased to 87.4% and 85.1% by the co-culture of miR-15a inhibitor and SC79, respectively (Fig. 8C). The number of migrated cells in the control, siR-NC+ inhibitor NC, siR-PFAR-2, siR-PFAR-2 + inhibitor, and siR-PFAR-2+ SC79 groups was 178.7, 182.0, 68.7, 112.0, and 113.7, respectively (Fig. 8D). Moreover, levels of RET, p-AKT/AKT, and p-mTOR/mTOR were notably decreased by siR-PFAR-2, which were sharply elevated in the siR-PFAR-2+ inhibitor and siR-PFAR-2+ SC79 group, respectively (Fig. 9).

## Discussion

Several researches have claimed the important role of LncRNAs in PTC. Lan (Lan et al. 2015) performed gene microarray analysis of 62 pairs of human PTC tissues and adjacent tissues, and found that 1192 LncRNAs were upregulated and 2307 LncRNAs were downregulated. GO analysis and signaling pathway analysis showed that the differentially expressed LncRNAs and miRNAs were related to cancer-related signaling pathways such as p53, MAPK, and PPAR, and 463 differentially expressed target genes may be regulated by LncRNAs. Cao (Cao et al. 2019) reported that 2925 LncRNAs were differentially expressed in 15 pairs of human PTC tissues and para-cancerous tissues, among which 1922 were upregulated and 933 were downregulated, indicating that LncRNA is an important regulator in PTC tumorigenesis. Sedaghati (Sedaghati and Kebebew 2019) summarized the dysregulated and functional LncRNAs in thyroid cancer, including 28 upregulated LncRNAs, such as ANRIL, BANCR, H19, HOTAIR, MALAT1, and NEAT1, and 22 downregulated LncRNAs, including GAS5, NAMA, PTCSC2, and PTCSC3. Herein, LncRNA PFAR was found markedly upregulated in PTC cell lines, implying a potential function of LncRNA PFAR in PTC. Although LncRNA PFAR is recently discovered and reported to be involved in several diseases, such as idiopathic pulmonary fibrosis (Zhao et al. 2018) and chronic pancreatitis (Zhang et al. 2021). The role of LncRNA PFAR malignant tumor was uncertain. Herein, to explore the potential function of LncRNA PFAR in PTC, in 4 PTC cell lines enrolled in the present study, LncRNA PFAR was knocked down in TPC-1 cells with the highest expression of LncRNA PFAR, while LncRNA PFAR was overexpressed in BCPAP cells with the lowest expression of LncRNA PFAR. Opposite results on the proliferation, migration, and apoptosis were observed between LncRNA PFAR-knockdown TPC-1 cells and LncRNA PFAR-overexpressed BCPAP cells, implying that LncRNA PFAR might exert an important oncogenic function in PTC.

In thyroid cancer tissues, the study by He and Pallante showed that miR-146b-5p, miR-221-3p and miR-222-3p were upregulated in PTC (He et al. 2005; Pallante et al. 2006). Zhang (Zhang et al. 2013) used gene chip technology to find that 10 miRNAs were upregulated and 5 miRNAs were downregulated in thyroid cancer, among which miR-146b-5p was the most significantly upregulated and miR-335 was the most significantly downregulated miRNA. The target genes of miRNA predicted by bioinformatics tools were involved in cell proliferation, differentiation, apoptosis, cell cycle, and signal transduction. These researches highlighted the important function of miRNAs in PTC. Herein, miR-15a was found to be a sponging miRNA of LncRNA PFAR, which is in line with data presented by Sun (Sun et al. 2019). Furthermore, the suppressive function of siR-PFAR-2 against the proliferation and migration of PTC cells was abolished by the inhibitor of miR-15a, implying a tumor suppressor function of miR-15a. In fact, miR-15a is previously found to be markedly downregulated in PTC (Hu et al. 2017). Moreover, miR-15a is reported to exert an inhibitory effect on PTC progression by regulating expressions of several proteins, including HOXA3 (Jiang et al. 2019) and AXIN2 (Zhao et al. 2015). These researches on the function of miR-15a in PTC were in accordance with data obtained in the present study.

RET is located on human chromosome 10 and can be cleaved into different forms of receptor tyrosine kinases such as RET9, RET43, and RET51, which constitute the single transmembrane tyrosine kinase receptor protein RET family (Li et al. 2019). Thyroid cancer is found closely related to the activation of RET. Studies have shown that RET tyrosine kinase inhibitors (vandetanib and cabozantinib) exert a significant effect on PTC (Paziewska et al. 2011; Carr et al. 2010; Pao and Girard 2011). Herein, RET was found to be targeted by miR-15a, which is in line with the research by Jin (Jin et al. 2019). RET is reported to participate in the development of malignant tumors by activating the AKT/mTOR signaling (Couto et al. 2012). Herein, LncRNA PFAR was found to signally activate the RET/AKT/mTOR signaling. Considering the interaction between LncRNA PFAR and miR-15a, as well as miR-15a and RET, we suspected that LncRNA PFAR sponged miR-15a to upregulate RET, which contributed to the activation of the AKT/mTOR signaling. Furthermore, the influence of siR-PFAR-2 on the proliferation and apoptosis of PTC cells was abolished by the inhibitor of AKT/mTOR signaling, which confirmed that LncRNA PFAR facilitated the progression of PTC by activating the AKT/mTOR signaling. In the future work, the involvement of RET in the regulatory function of LncRNA PFAR against PTC will be further confirmed by treating RET-knockdown PTC cells with the pcDNA3.1-PFAR. In addition, the prognosis value of LncRNA PFAR in patients with PTC will be analyzed in our future work to further confirm the biofunction of LncRNA PFAR. Furthermore, there are limitations in the present study. Compared to BCPAP cells, LncRNA PFAR was not knocked down in the Nthy-ori3-1 cell line, and the effect of LncRNA PFAR downregulation on proliferation, apoptosis, migration, invasion in normal thyroid gland epithelial cells was not observed, which should be further studied in our future work. In addition, RET mutation is previously reported in TPC-1 cells (Gilbert-Sirieix et al. 2010). Considering the negative control (siR-NC and inhibitor NC) utilized in the present study, the influence of RET mutation on the miR-15a/RET axis in TPC-1 cells has been narrowed down. Moreover, it is reported that RET mutation has not been observed in BCPAP cells (Meireles et al. 2007). In BCPAP cells the influence of RET mutation on the miR-15a/RET axis was excluded.

Collectively, LncRNA PFAR facilitated the proliferation and migration of PTC cells by mediating the miR-15a/RET axis.

### Supplementary Information

Below is the link to the electronic supplementary material.

Supplementary file1 (JPG 900 KB)

## Data Availability

All data are available from the corresponding author if requested by the journal or the readers.

## References

[CR1] Asa SL (2019). The current histologic classification of thyroid cancer. Endocrinol Metab Clin North Am.

[CR2] Bridges MC, Daulagala AC, Kourtidis A (2021) LNCcation: lncRNA localization and function. J Cell Biol 220(2):e202009045 10.1083/jcb.202009045 PMC7816648 33464299

[CR3] Cao Y, Shi C, Li J, Liang Y, Qiu J, Yuan L, Yong Z, Zhang DX, Shi GQ (2019). Expression profile of long noncoding RNAs in human papillary thyroid carcinoma. Neoplasma.

[CR4] Carr LL, Mankoff DA, Goulart BH, Eaton KD, Capell PT, Kell EM, Bauman JE, Martins RG (2010). Phase II study of daily sunitinib in FDG-PET-positive, iodine-refractory differentiated thyroid cancer and metastatic medullary carcinoma of the thyroid with functional imaging correlation. Clin Cancer Res.

[CR5] Couto JP, Almeida A, Daly L, Sobrinho-Simoes M, Bromberg JF, Soares P (2012). AZD1480 blocks growth and tumorigenesis of RET- activated thyroid cancer cell lines. PLoS ONE.

[CR6] Entezari M, Ghanbarirad M, Taheriazam A, Sadrkhanloo M, Zabolian A, Goharrizi M, Hushmandi K, Aref AR, Ashrafizadeh M, Zarrabi A, Nabavi N, Rabiee N, Hashemi M, Samarghandian S (2022). Long non-coding RNAs and exosomal lncRNAs: Potential functions in lung cancer progression, drug resistance and tumor microenvironment remodeling. Biomed Pharmacother.

[CR7] Gierlikowski W, Gierlikowska B (2022). MicroRNAs as regulators of phagocytosis. Cells.

[CR8] Gilbert-Sirieix M, Ripoche H, Malvy C, Massaad-Massade L (2010). Effects of silencing RET/PTC1 junction oncogene in human papillary thyroid carcinoma cells. Thyroid.

[CR9] Haddad RI, Bischoff L, Ball D, Bernet V, Blomain E, Busaidy NL, Campbell M, Dickson P, Duh QY, Ehya H, Goldner WS, Guo T, Haymart M, Holt S, Hunt JP, Iagaru A, Kandeel F, Lamonica DM, Mandel S, Markovina S, McIver B, Raeburn CD, Rezaee R, Ridge JA, Roth MY, Scheri RP, Shah JP, Sipos JA, Sippel R, Sturgeon C, Wang TN, Wirth LJ, Wong RJ, Yeh M, Cassara CJ, Darlow S (2022) Thyroid carcinoma, version 2.2022, NCCN Clinical Practice Guidelines in Oncology. J Natl Compr Canc Netw 20(8):925–951 10.6004/jnccn.2022.0040 35948029

[CR10] He H, Jazdzewski K, Li W, Liyanarachchi S, Nagy R, Volinia S, Calin GA, Liu CG, Franssila K, Suster S, Kloos RT, Croce CM, de la Chapelle A (2005). The role of microRNA genes in papillary thyroid carcinoma. Proc Natl Acad Sci U S A.

[CR11] Hu J, Li C, Liu C, Zhao S, Wang Y, Fu Z (2017). Expressions of miRNAs in papillary thyroid carcinoma and their associations with the clinical characteristics of PTC. Cancer Biomark.

[CR12] Hu J, Yuan IJ, Mirshahidi S, Simental A, Lee SC, Yuan X (2021) Thyroid carcinoma: phenotypic features, underlying biology and potential relevance for targeting therapy. Int J Mol Sci 22(4):1950 10.3390/ijms22041950 PMC7920269 33669363

[CR13] Jiang L, Wu Z, Meng X, Chu X, Huang H, Xu C (2019). LncRNA HOXA-AS2 facilitates tumorigenesis and progression of papillary thyroid cancer by modulating the miR-15a-5p/HOXA3 axis. Hum Gene Ther.

[CR14] Jin J, Zhang J, Xue Y, Luo L, Wang S, Tian H (2019). miRNA-15a regulates the proliferation and apoptosis of papillary thyroid carcinoma via regulating AKT pathway. Onco Targets Ther.

[CR15] Jonas S, Izaurralde E (2015). Towards a molecular understanding of microRNA-mediated gene silencing. Nat Rev Genet.

[CR16] Komatsu S, Kitai H, Suzuki HI (2023). Network regulation of microRNA biogenesis and target interaction. Cells.

[CR17] Lan X, Zhang H, Wang Z, Dong W, Sun W, Shao L, Zhang T, Zhang D (2015). Genome-wide analysis of long noncoding RNA expression profile in papillary thyroid carcinoma. Gene.

[CR18] Li AY, McCusker MG, Russo A, Scilla KA, Gittens A, Arensmeyer K, Mehra R, Adamo V, Rolfo C (2019). RET fusions in solid tumors. Cancer Treat Rev.

[CR19] Lin W, Zhou Q, Wang CQ, Zhu L, Bi C, Zhang S, Wang X, Jin H (2020). LncRNAs regulate metabolism in cancer. Int J Biol Sci.

[CR20] Mahmoudian-Sani MR, Jalali A, Jamshidi M, Moridi H, Alghasi A, Shojaeian A, Mobini GR (2019). Long non-coding RNAs in thyroid cancer: implications for pathogenesis diagnosis, and therapy. Oncol Res Treat.

[CR21] Meireles AM, Preto A, Rocha AS, Rebocho AP, Maximo V, Pereira-Castro I, Moreira S, Feijao T, Botelho T, Marques R, Trovisco V, Cirnes L, Alves C, Velho S, Soares P, Sobrinho-Simoes M (2007). Molecular and genotypic characterization of human thyroid follicular cell carcinoma-derived cell lines. Thyroid.

[CR22] Pallante P, Visone R, Ferracin M, Ferraro A, Berlingieri MT, Troncone G, Chiappetta G, Liu CG, Santoro M, Negrini M, Croce CM, Fusco A (2006). MicroRNA deregulation in human thyroid papillary carcinomas. Endocr Relat Cancer.

[CR23] Pao W, Girard N (2011). New driver mutations in non-small-cell lung cancer. Lancet Oncol.

[CR24] Park EG, Pyo SJ, Cui Y, Yoon SH, Nam JW (2022) Tumor immune microenvironment lncRNAs. Brief Bioinform 23(1):bbab504 10.1093/bib/bbab504 PMC8769899 34891154

[CR25] Paziewska A, Harris PD, Zwolinska L, Bajer A, Sinski E (2011). Recombination within and between species of the alpha proteobacterium Bartonella infecting rodents. Microb Ecol.

[CR26] Pinto A, Morello S, Sorrentino R (2011). Lung cancer and Toll-like receptors. Cancer Immunol Immunother.

[CR27] Pozniak T, Shcharbin D, Bryszewska M (2022). Circulating microRNAs in medicine. Int J Mol Sci.

[CR28] Sedaghati M, Kebebew E (2019). Long noncoding RNAs in thyroid cancer. Curr Opin Endocrinol Diabetes Obes.

[CR29] Sun J, Su W, Zhao X, Shan T, Jin T, Guo Y, Li C, Li R, Zhou Y, Shan H, Sun X, Liang H (2019) LncRNA PFAR contributes to fibrogenesis in lung fibroblasts through competitively binding to miR-15a. Biosci Rep 39(7):BSR20190280 10.1042/BSR20190280 PMC6639460 31273058

[CR30] Tong R, Zhang J, Wang C, Li X, Yu T, Wang L (2020). LncRNA PTCSC3 inhibits the proliferation, invasion and migration of cervical cancer cells via sponging miR-574-5p. Clin Exp Pharmacol Physiol.

[CR31] Wang Z, Zhang H, He L, Dong W, Li J, Shan Z, Teng W (2013). Association between the expression of four upregulated miRNAs and extrathyroidal invasion in papillary thyroid carcinoma. Onco Targets Ther.

[CR32] Wei C, Song H, Sun X, Li D, Song J, Hua K, Fang L (2015). miR-183 regulates biological behavior in papillary thyroid carcinoma by targeting the programmed cell death 4. Oncol Rep.

[CR33] Xu J, Zhang Y, You Q, Fu H, Zhao X, Lu K, Yan R, Yang D (2020). LncRNA PTCSC3 alleviates the postoperative distant recurrence of gastric cancer by suppression of lncRNA HOXA11-AS. Cancer Manag Res.

[CR34] Yang J, Liu F, Wang Y, Qu L, Lin A (2022). LncRNAs in tumor metabolic reprogramming and immune microenvironment remodeling. Cancer Lett.

[CR35] Zhang B, Pan X, Cobb GP, Anderson TA (2007). microRNAs as oncogenes and tumor suppressors. Dev Biol.

[CR36] Zhang J, Liu Y, Liu Z, Wang XM, Yin DT, Zheng LL, Zhang DY, Lu XB (2013). Differential expression profiling and functional analysis of microRNAs through stage I-III papillary thyroid carcinoma. Int J Med Sci.

[CR37] Zhang J, Yang Y, Liu Y, Fan Y, Liu Z, Wang X, Yuan Q, Yin Y, Yu J, Zhu M, Zheng J, Lu X (2014). MicroRNA-21 regulates biological behaviors in papillary thyroid carcinoma by targeting programmed cell death 4. J Surg Res.

[CR38] Zhang G, Chi N, Lu Q, Zhu D, Zhuang Y (2020). LncRNA PTCSC3 is a biomarker for the treatment and prognosis of gastric cancer. Cancer Biother Radiopharm.

[CR39] Zhang T, Zhang G, Yang W, Chen H, Hu J, Zhao Z, Cheng C, Li G, Xie Y, Li Y, Kong R, Wang Y, Wang G, Chen H, Bai XW, Pan S, Sun B, Li L (2021). Lnc-PFAR facilitates autophagy and exacerbates pancreatic fibrosis by reducing pre-miR-141 maturation in chronic pancreatitis. Cell Death Dis.

[CR40] Zhao Y, Liu X, Zhong L, He M, Chen S, Wang T, Ma S (2015). The combined use of miRNAs and mRNAs as biomarkers for the diagnosis of papillary thyroid carcinoma. Int J Mol Med.

[CR41] Zhao X, Sun J, Chen Y, Su W, Shan H, Li Y, Wang Y, Zheng N, Shan H, Liang H (2018). lncRNA PFAR promotes lung fibroblast activation and fibrosis by targeting miR-138 to regulate the YAP1-Twist axis. Mol Ther.

[CR42] Zhao Y, Wei D, Zhang Y, Ji J (2022). Panoramic view of microRNAs in regulating cancer stem cells. Essays Biochem.

[CR43] Zheng H, Wang M, Jiang L, Chu H, Hu J, Ning J, Li B, Wang D, Xu J (2016). BRAF-activated long noncoding RNA modulates papillary thyroid carcinoma cell proliferation through regulating thyroid stimulating hormone receptor. Cancer Res Treat.

