# Supplementary figures and images for: LncRNA PFAR facilitates the proliferation and migration of papillary thyroid carcinoma by competitively binding to miR-15a
Source: Naunyn Schmiedebergs Arch Pharmacol. 2023 Oct 24;397(5):3037–48. doi: 10.1007/s00210-023-02779-w (PMC11074224; doi:10.1007/s00210-023-02779-w)

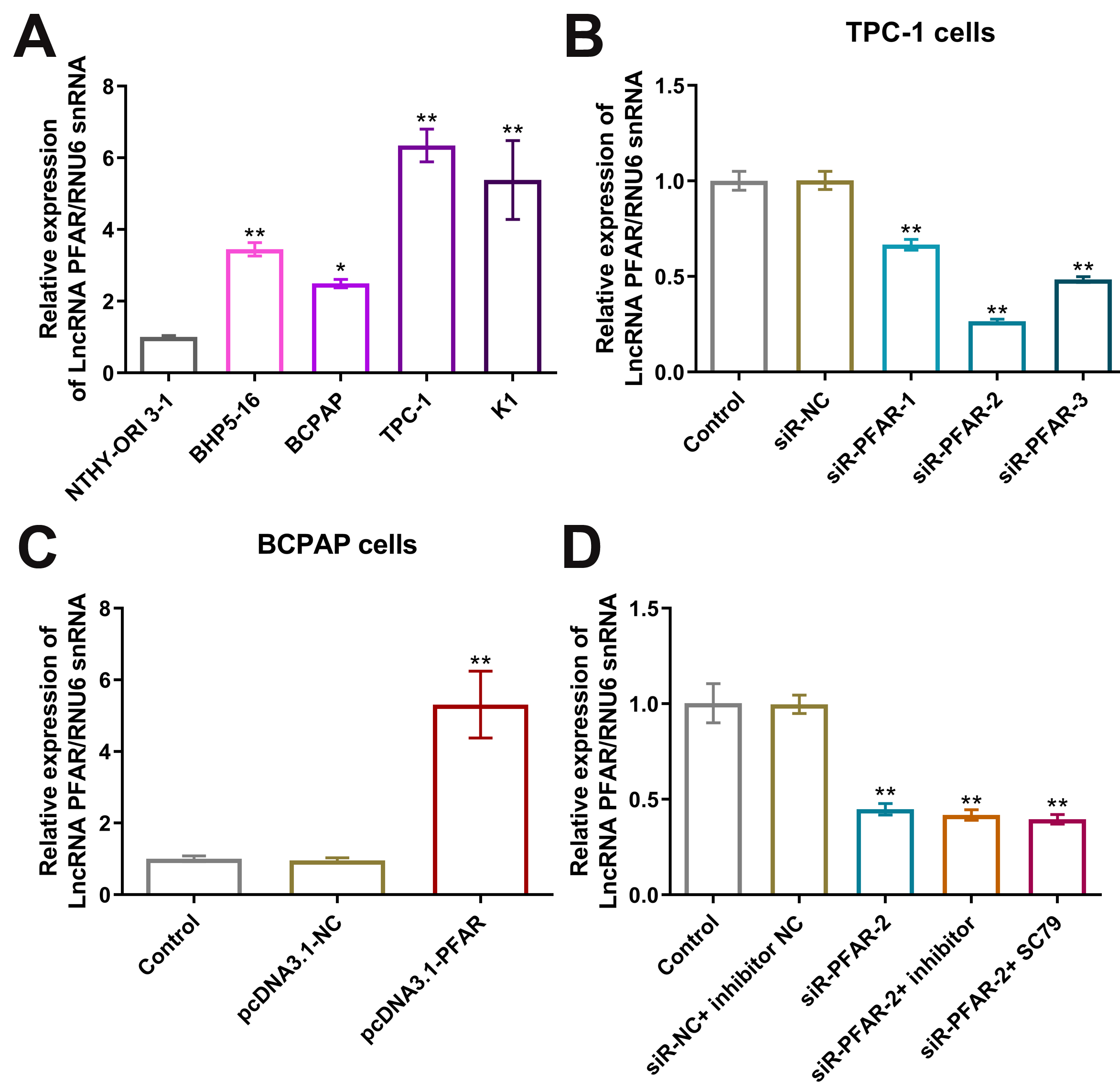

Supplement: Supplementary file 1 — Supplementary file1 (JPG 900 KB) [file 210_2023_2779_MOESM1_ESM.jpg]
